# Just Not Enough: Utilization of Outpatient Psychotherapy Provided by Clinical Psychologists for Patients With Psychosis and Bipolar Disorder in Switzerland

**DOI:** 10.1177/11786329241229950

**Published:** 2024-02-11

**Authors:** Mariela E Jaffé, Sou Bouy Loew, Andrea H Meyer, Roselind Lieb, Frieder Dechent, Undine E Lang, Christian G Huber, Julian Moeller

**Affiliations:** 1 University Psychiatric Clinics (UPK), University of Basel, Basel, Switzerland; 2 Division of Clinical Psychology and Epidemiology, Department of Psychology, University of Basel, Basel, Switzerland

**Keywords:** Psychosis, schizophrenia, psychotherapy, outpatient, utilization

## Abstract

Treatment guidelines state that evidence-based psychotherapy is effective for people with psychosis and bipolar disorder and should be offered during every phase of the treatment process. However, research has indicated a lack of outpatient psychotherapeutic services for this patient group, for example, in the United States or Germany. We extend this finding by presenting survey data from Switzerland. We surveyed 112 inpatients with a diagnosis of a schizophrenia spectrum disorder or bipolar disorder and assessed outpatient treatment over the 5 years prior to their index hospitalization by using retrospective self-reports. The survey focused on psychotherapy provided by clinical psychologists. Results indicate that only 23.2% of participants retrospectively reported having utilized any outpatient psychotherapy within the reporting period and only 8% of participants reported having received a number of outpatient sessions that reaches recommended levels of psychotherapy. Exploratory analyses did not detect a significant association between self-reported utilization of outpatient psychotherapy sessions and most demographic, psychiatric, and psychological attributes, but patients with a bipolar disorder diagnosis (vs schizophrenia spectrum diagnosis) reported having utilized outpatient treatment more often. These findings are preliminary. When replicated they highlight the need for increased access to outpatient psychotherapy and better alignment between guideline recommendations and outpatient supply.

## Background

Current treatment guidelines delineate that evidence-based psychotherapy should be offered to all people with psychosis, throughout all phases of the treatment process, see NICE-or S3-Guidelines^1,2^; for the recommendations by the American Psychiatric Association.^3^ Both, NICE-^1^ and S3-Guidelines^2^ recommend cognitive behavioral therapy (CBT, and family interventions) explicitly, as well as other approaches (eg, arts therapy, or metacognitive training, psychodynamic therapy, body and movement based therapy, and social skill training). More specifically, the guidelines also specify the intensity of treatment that should be provided by healthcare professionals, identifying more than 15 sessions as sufficient treatment, yet recommending more than 24 sessions in evidence-based psychotherapy for people with psychosis.^2^ Recommendations for psychotherapy also hold true for bipolar disorder, see, for example, the NICE-Guidelines^4^ or the guidelines by the American Psychiatric Association.^5^ To foster the recommended utilization of evidence-based psychotherapy by people with psychosis and bipolar disorder, inpatient and outpatient treatment such as CBT needs to be available, accessible, and ultimately delivered.

Research, however, indicates a substantial undersupply of psychotherapy for people with psychosis^6-9^ and bipolar disorder^10,11^ and a gap between guideline recommendations and utilization.

### Utilization of cognitive behavioral therapy in different countries

When looking at previous research from the *United Kingdom*, average implementation rates of CBT for patients with psychosis are estimated to be 46% with high variance in estimates and problems concerning the distinction between services that are offered, delivered, or received^6^ (p. 329). When looking at smaller, more controlled studies, this number drops to 6.9% of patients having been offered CBT and 5.3% of patients who actually utilized CBT, cf.^12^

Furthermore, Colling et al,^8^ analyzed data from a large electronic health record database. They used natural language processing to estimate how many service users in the database were diagnosed with a schizophrenia spectrum disorder and for how many of these users the receipt of CBT for psychosis (CBTp) sessions was recorded in the health record. Overall, 34.6% of service users had received at least 1 CBTp session and 26.4% at least 2 CBTp sessions, still leaving a large proportion of patients without any CBTp sessions. A utilization of 1 or 2 CBT sessions, however, does not align with current guideline recommendations. Stefanova et al^13^ replicate this finding and also indicate low numbers of receipt of evidence-based therapy sessions (both CBTp and/or family interventions) in their study.

When turning to the *USA*, researchers, such as Kopelovich et al,^14^ have again identified a substantial gap between the need for CBTp and the available supply. They also indicate a lack of research on its implementation and dissemination across the USA,^15^ see also Kopelovich et al.^16^ Based on current training levels of healthcare professionals, the authors estimate that only 0.3% of 5 million people with a primary psychotic disorder utilize CBTp^14^ (p. 755).

For *Germany*, Schlier and Lincoln^7^ describe data across 3 studies and report that about 50% of patients with schizophrenia spectrum disorders in inpatient psychiatric hospitals have utilized general psychological treatment. The proportion of patients, however, who received cognitive and/or behavioral therapy in a one-on-one setting was low (numbers range between 5.5%-6.3% for cognitive therapy and 4.4%-8.0% for behavioral therapy). The authors summarize that the majority of inpatients therefore have not utilized CBT that is in line with the respective guideline recommendations (S3).

When looking at bipolar disorders in particular, data from *Italy* paints a similar picture. Barbato et al^10^ showed that the rate of patients with bipolar disorder who utilized individual psychotherapy was estimated at 6.1%. Later research^11^ estimated the use of psychotherapy at 11.5% for people with bipolar disorders, but the authors also highlight that treatment was characterized by low intensity.

### Exploring potential correlates of utilization of evidence-based therapy

Whereas the previously summarized research investigated the overall provision and utilization of evidence-based treatment such as CBT, other research has examined patients’ attributes that may be associated with utilization of psychotherapy among people with psychosis and bipolar disorder. In general, different research has identified a heterogeneous picture of different demographic (eg, gender, age, or nationality), psychiatric (eg, symptom severity), and psychological (eg, self-esteem or motivation) attributes that may be associated with the utilization of treatment. For an overview on associations between attributes and general treatment utilization, see, Lo et al.^17^

Stefanova et al,^13^ for example, focused specifically on persons with psychosis and investigated who utilized evidence-based therapy (CBTp or family interventions) after a hospital admission. Importantly, they focused on a sample of service users that had already demonstrated a willingness to engage in psychotherapy. Their results indicated that utilization of evidence-based therapy was more likely for participants with lower delusional distress, a Black and Minority Ethnic background, and when participants were discharged to an early intervention service. This research showed that a mix of demographic (such as ethnic background) and psychiatric (such as delusions) attributes were associated with treatment utilization, even when controlling for psychological attributes (eg, treatment motivation).

In a different line of work, Harvey et al^18^ assessed demographic (eg, age, gender, education), functional (eg, disabilities), mental and physical health characteristics as well as previous service use and then analyzed potential associations with self-reported utilization of evidence-based therapy, in which they included CBTp, family psychoeducation, relapse prevention planning, skills training, supported employment, and assertive community treatment. Results indicated that demographic attributes such as gender and age as well as psychological/psychiatric attributes such as insight into the disorder were significantly associated with the utilization of evidence-based treatment. Attributes regarding previous service utilization and characteristics (eg, community treatment in the past, availability of case managers in different disciplines), however, were more predictive in the model.

Overall, previous studies identified heterogeneous demographic factors, psychological attributes and treatment motivation, as well as psychiatric aspects such as symptom severity and previous treatment history/experience as relevant factors when it comes to utilization of psychotherapy. Further research, however, is required to provide a harmonized meta-model summarizing correlates of utilization of psychotherapy for people with psychosis and bipolar disorder.

### A perspective on the situation in Switzerland

In Switzerland, psychotherapy can be provided by qualified physicians or clinical psychologists may provide psychotherapy if they are currently enrolled or have completed specific, comprehensive postgraduate training. Psychiatric-psychotherapeutic treatment offered by psychiatrists includes, for example, medical consultations, pharmacotherapy, and psychotherapy in the narrow sense,^19^ whereas clinical psychologists focus on psychotherapy only. Both, qualified physicians and clinical psychologists, therefore, shape the outpatient treatment landscape. It is noteworthy, however, that in the outpatient network, psychiatrists or clinical psychologists may decide which patients with which syndromes/diagnoses they accept into treatment. Treatment costs, in general, are covered by the mandatory health insurance (up to a certain number of sessions).

While an underutilization of psychotherapy among people with psychosis and bipolar disorder was reported for both the inpatient and outpatient setting in the UK, USA, Germany, and Italy, there is little research on this topic in Switzerland. It remains unclear which patient attributes are associated with the utilization of psychotherapy in Switzerland. This represents a research gap, as it is unclear whether the findings described above can be generalized to other countries with different health care systems. Reports suggest that differences in health care systems in general (across Europe, see, Wendt^20^) and mental health care systems in particular are present.^21,22^ The current discourse aiming to improve alignment and resource allocation in psychiatric and psychotherapeutic services in Switzerland, furthermore, highlights the relevance of gaining a better understanding of the utilization of mental health services.^23,24^

In this manuscript, we report data from a catchment area in the German-speaking part of Switzerland. We outline a cross-sectional survey in which we collected data from 112 inpatients with psychosis and bipolar disorder and analyzed data covering a reporting period between 1 and 5 years preceding their index stay at an inpatient psychiatric clinic. In this project, we investigated (a) the presence or absence of retrospectively self-reported utilization of outpatient psychotherapy and (b) whether the utilized number of outpatient psychotherapy sessions was in line with current guideline recommendations. Last, we aim to explore potential differences in patients who retrospectively self-report having utilized psychotherapy with clinical psychologists versus those who report not having utilized it by looking at associations between demographic, psychiatric, and psychological attributes as well as the reported presence or absence of utilization of outpatient psychotherapy during the reporting period. Based on previous research, we here focused on attributes such as gender, age, and education (demographic, see Lo et al^17^), the respective diagnosis, psychiatric symptoms, previous (involuntary) hospitalizations as a proxy for treatment history/experience (psychiatric, see Lo et al^17^ and Harvey et al^18^), and self-esteem, self-efficacy, and attitudes regarding psychotherapy (psychological, see Stefanova et al^13^ and Lo et al^17^).

## Methods

We collected the cross-sectional study data presented in this manuscript within a project on high utilization patterns among psychiatric inpatients. The full study background and methods were described by Lo et al.^17^ The Ethics Committee Northwest and Central Switzerland (EKNZ) approved this study (BASEC 2917-02203) and the research protocol adheres to the principles outlined in the Declaration of Helsinki. The study was conducted between April 2018 and May 2019 and therefore provides a pre-COVID-19 outlook on the utilization of outpatient psychotherapy for patients with psychosis and bipolar disorder.

### Participants and data

As participants we recruited inpatients with schizophrenia spectrum disorder (ICD-10: F20-F29) or bipolar affective disorder (ICD-10: F31.1, F31.2) at the end of their inpatient treatment at the Centre for Psychotic Disorders at the University Psychiatric Hospital (UPK) in Basel, Switzerland. If inpatients agreed to participate in the study, a psychologist conducted the assessment, see Lo et al.^17^ The total sample consists of 112 participants (see Results section below).

### Measures and procedure

Assessments included interviews, paper-pencil-questionnaires, and information retrieved from electronic patient records. Information on the utilization of treatment was collected with slightly modified versions of the Client Sociodemographic and Service Receipt Inventory (short CSSRI,^25^) and included the retrospectively self-reported number of psychotherapy sessions with clinical psychologists in the last 5 years (also specifying the number of sessions in the last 12 months). We focused on clinical psychologists, as they provide psychotherapy without any medical consultation or pharmacotherapy, allowing us to estimate how much psychotherapy in the narrow sense has been utilized by the participants (see also Tadmon and Olfson^26^ for a longitudinal outlook on changing treatment patterns among psychiatrists in the US, indicating lower rates of psychotherapy over time). To complement these numbers, we further assessed the retrospectively self-reported number of sessions with psychiatrists in the last 12 months preceding the index stay. We did not ask participants whether the sessions with a psychiatrist had a psychiatric or psychotherapeutic focus, as we assumed that estimates would have been vague due to the limits of self-report and the retrospective nature of the study.

When patients reported having utilized outpatient psychotherapy, we asked them for details about the respective provider to identify whether the sessions were provided in-house (eg, in the specialized outpatient clinic of the UPK) or outside of the clinic. We furthermore aimed to analyze the provider’s training background in psychotherapy using external sources (eg, their website).

To better understand potential differences in patients who retrospectively reported (not) having utilized psychotherapy, we further investigated demographic, psychiatric, and psychological attributes of the patients. All demographic attributes (gender, age, education) were retrieved from the patients’ electronic records. For the psychiatric attributes, we assessed the extent of psychiatric symptoms using the Brief Psychiatric Rating Scale^27^ in its German translation.^28^ The patients’ primary diagnosis, year of the first stay in an inpatient clinic, the number of inpatient stays within the last 30 months (see Lo et al^17^), and whether patients were admitted (in)voluntarily to the psychiatric clinic were gathered from the patients’ electronic records. Regarding the psychological attributes, self-esteem was measured with the Rosenberg Self-Esteem Scale by Rosenberg^29^ (p. 291). In our study, we used a slightly adapted version of the German translation by Ferring and Filipp.^30^ Self-efficacy was measured with the General Self-Efficacy Scale by Schwarzer and Jerusalem.^31^ To assess attitudes toward psychotherapy, we used a subscale from the questionnaire on attitudes toward psychotherapeutic treatment.^32^ As recommended in later work,^33,34^ we used the positive attitudes toward psychotherapy subscale in our study. To assess hope in the context of psychotherapy, we integrated the respective subscale from the therapy motivation questionnaire^35^; items have been slightly rephrased.

### Data preparation and statistical analyses

Considering the statistical analyses, the first set of analyses focused on descriptively characterizing patients’ retrospectively self-reported utilization of outpatient psychotherapy supplied by a clinical psychologist. In the second set of analyses, we used bivariate regression analyses to examine associations between demographic, psychiatric, or psychological patient characteristics and their retrospectively self-reported utilization of outpatient psychotherapy provided by clinical psychologists. As the target variable was binary (1 = *utilization of at least 1 session of psychological psychotherapy within the last 5* *years*; 0 = *no session*), we computed logistic regression models. All statistical correlates in the regression models were *z*-standardized (in the case of continuous variables such as age, BPRS score, number of stays in the psychiatric clinic, first stay in a psychiatric clinic, self-esteem, self-efficacy, positive attitude toward psychotherapy, and motivation for psychotherapy/hope) or effect coded (for categorical variables such as gender, diagnosis, and involuntary hospitalization; with −0.5 = *involuntary admission/F20 diagnosis/male participants* and 0.5 = *voluntary admission/F31 diagnosis/female participants*). The attribute education had 3 levels, which were coded as −1 = *mandatory education*, 0 = *secondary level education*, and 1 = *high school equivalent*.

In regards to all statistical analyses, it is important to highlight that this study was designed as a cross-sectional survey in which all variables were assessed at the same time. The analyses were computed using RStudio, version 2022.02.1.^36^

## Results

### Descriptive Results

One hundred twelve patients participated in the study (62 male, 50 female; *M*_age_ = 40.40, *SD*_age_ = 12.04; 86 with an F2X diagnosis and 26 with an F31 diagnosis). Several of them had previously completed multiple inpatient stays in the psychiatric clinic, with an average of 3.76 stays within the last 30 months. When looking at the index stay, 83 participants (74.11%) entered the clinic voluntarily, whereas 29 (25.89%) entered with a compulsory admission; for further information on the sample, see Table 2 or Lo et al.^17^

**Figure 1.**
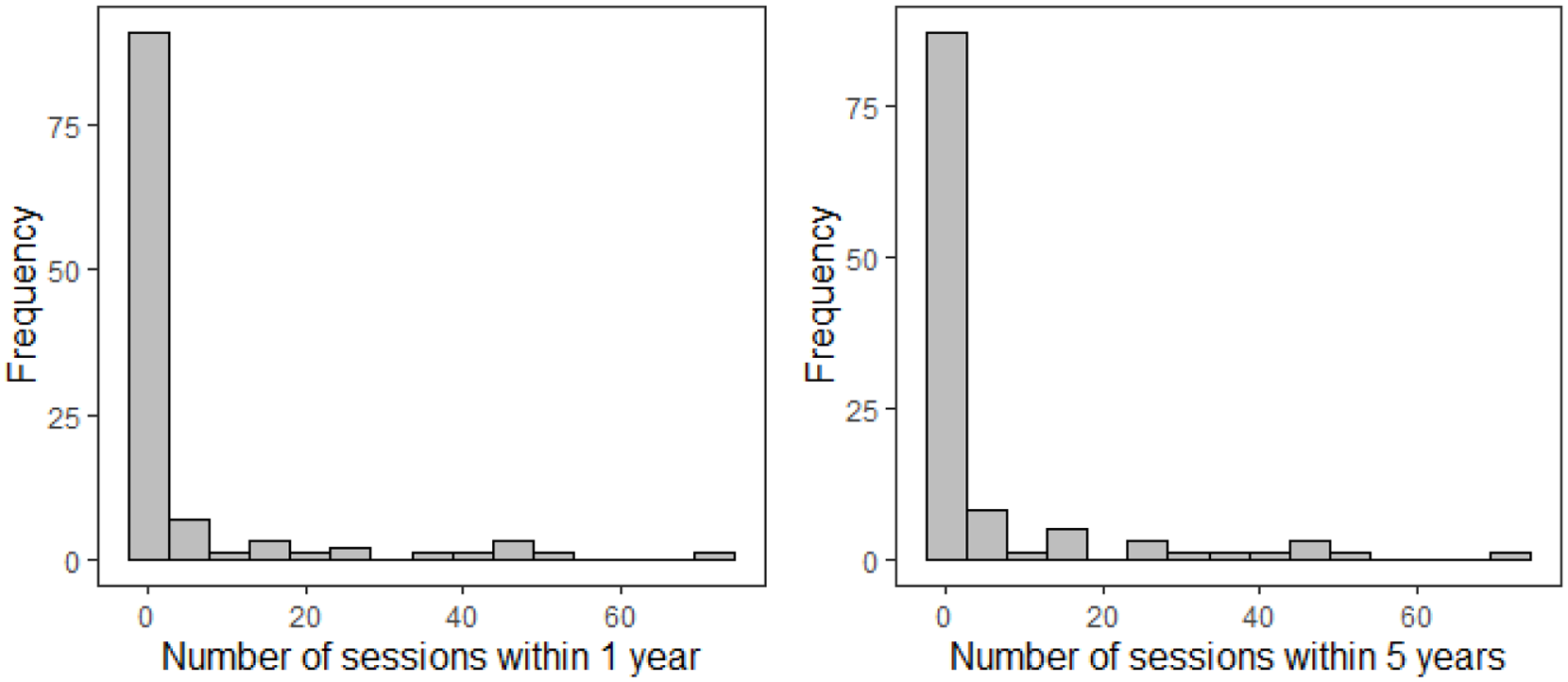
Patients’ retrospectively self-reported number of outpatient psychotherapy sessions with a psychologist in the last year and last 5 years preceding the index stay at the psychiatric hospital.

When looking at descriptive results, participants on average retrospectively reported having utilized 4.51 outpatient psychotherapy sessions with clinical psychologists within the past 12 months. When extending the period to the last 5 years, the number of reported outpatient psychotherapy sessions with psychologists only slightly increased to 5.16 sessions (see Table 1, and also Figure 1 for a visual depiction of the distribution). In-house clinical psychologists provided the majority of these sessions (see Table 1). Only 19.6% (n = 22) of the patients reported having utilized at least 1 outpatient session with a clinical psychologist within the last 12 months and 23.2% (n = 26) within the last 5 years.

**Table 1.** Summary of descriptive results on patients’ retrospectively self-reported outpatient treatment utilization in the last year and last 5 years preceding the index stay at the psychiatric hospital.

Number of sessions with	Range	*M*	25% *Q*	Md	75% *Q*
Psychologists (1 y)	0-72	4.51	0.00	0.00	0.00
. . . within the psychiatric hospital (in-house UPK)	0-48	3.06	0.00	0.00	0.00
. . . external	0-72	1.45	0.00	0.00	0.00
Psychologists (5 y)	0-72	5.16	0.00	0.00	0.00
. . . within the psychiatric hospital (in-house UPK)	0-48	3.20	0.00	0.00	0.00
. . . external	0-72	1.96	0.00	0.00	0.00
Psychiatrists (1 y)	0-84	11.56	0.00	5.00	17.75
. . . within the psychiatric hospital or at the outpatient clinic (in-house UPK)	0-48	2.00	0.00	0.00	0.00
. . . external	0-84	9.56	0.00	0.00	12.00

Even though some of the sessions were supplied by the psychiatric hospital (in-house UPK), these contribute to the number of outpatient treatment sessions, as they took place outside of an inpatient stay.

Patients also named the clinical psychologists who provided the outpatient psychotherapy sessions. In total 22 (3 not identified) different psychologists provided psychotherapy to the 26 patients over the 5-year period. Of these 22 psychologists, 9 had a cognitive behavioral background, 3 a behavioral background, 3 a system or solution-focused background, 3 a psychoanalytic background, and for 4 psychotherapists the background was unknown.

We further compared the number of patients’ retrospectively self-reported utilized outpatient psychotherapy sessions provided by a clinical psychologist in the 5 years preceding the index stay with the number of sessions recommended by current guidelines. Results indicate that 12.5% of participants (*n* = 14) reported having utilized at least sufficient psychotherapeutic treatment (more than 15 sessions, see S3-Guidelines^2^). Moreover, 8.0% (*n* = 9) reported having utilized at least the number of sessions that are considered as recommended treatment (more than 24 sessions, see S3-Guidelines^2^).

To allow comparisons with outpatient sessions utilized with a psychiatrist (that may cover psychiatric and medical content as well as psychotherapy), we report the respective number of sessions; however, the numbers were only surveyed for the 12 months prior to the index stay. Participants indicated having utilized on average 11.56 outpatient sessions with psychiatrists in the last 12 months, mostly provided by external psychiatrists (outside of UPK, see Table 1). Looking at the distribution of sessions, 63.4% (*n* = 71) reported at least 1 outpatient session with a psychiatrist within the last 12 months.

### Correlates of utilization of psychotherapy for psychosis

We explored associations between participants’ demographic, psychiatric, and psychological attributes, and whether (or not) they retrospectively reported at least 1 outpatient therapy session with a clinical psychologist within the last 5 years. We computed bivariate logistic regression models as overfitting has been reported to be a problem for multiple regression, see, for example, James et al,^37^ especially when the model is based on many predictor variables. Results are summarized in Table 2 and indicate that the primary diagnosis (F2X vs F31) was associated with the self-report of utilization of outpatient psychotherapy. The odds ratio of 2.73 indicates that patients with a F31 diagnosis were more likely to report psychological outpatient psychotherapy compared to patients with F2X diagnoses. Descriptive results indicate that 10 of the 26 patients with an F31 diagnosis reported having at least 1 psychotherapy session with a clinical psychologist in the last 5 years prior to the index stay (38.46%), whereas this was true for only 16 out of 86 patients with an F2X diagnosis (18.60%).

**Table 2.** Descriptive results for all attributes, complemented by results from bivariate logistic regression models looking at associations between attributes and retrospectively self-reported utilization of psychotherapy provided by a clinical psychologist.

			Utilization of therapy provided by a clinical psychologist within last 5 y				Target variable from the bivariate logistic regression model		
	Overall		Yes		No				
Attribute	*M*/n	SD	*M*/n	SD	*M*/n	SD	*b (SE b) P*	OR	95% CI
Gender	62 Male, 50 female		13 Male, 13 female		49 Male, 37 female		0.28 (0.45) .531	1.32	0.55-3.21
Age	40.40	12.04	35.85	10.72	41.78	12.13	–0.53 (0.24) .031	0.59	0.36-0.94
Education	37 Mandatory		8 Mandatory		29 Mandatory		0.41 (0.30) .174	1.51	0.84-2.78
	49 Secondary		8 Secondary		41 Secondary				
	26 High school equ.		10 High school equ.		16 High school equ.				
BPRS	41.84	10.65	43.38	10.62	41.37	10.68	0.19 (0.22) .398	1.21	0.78-1.89
Number of stays in inpatient clinic within 30 mo	3.76	3.73	4.50	3.47	3.53	3.80	0.23 (0.21) .264	1.26	0.83-1.92
First stay in inpatient clinic (year)	2004.28	11.52	2008.46	9.52	2003.01	11.82	0.53 (0.25) .038	1.70	1.05-2.88
F2X vs F31 diagnosis	86 F2X, 26 F31		16 F2X, 10 F31		70 F2X, 16 F31		1.01 (0.49) .040	2.73	1.03-7.14
Involuntary hospitalization	29 Yes, 83 no		7 Yes, 19 no		22 Yes, 64 no		–0.07 (0.51) .891	0.93	0.36-2.66
Self–esteem	38.02	8.11	38.19	7.50	37.97	8.32	0.03 (0.23) .900	1.03	0.66-1.62
Self–efficacy	29.03	6.60	28.08	6.16	29.31	6.74	–0.19 (0.22) .402	0.83	0.53–1.29
Positive attitude toward psychotherapy	17.96	4.59	18.42	4.60	17.83	4.61	0.13 (0.23) .560	1.14	0.74-1.84
Motivation for psychotherapy/hope	23.81	4.42	23.46	4.29	23.92	4.48	–0.10 (0.22) .643	0.90	0.59-1.41

To a lesser extent (given the smaller effect sizes), age and the first stay in the inpatient clinic appeared to show an association with the retrospectively self-reported utilization of psychotherapy provided by clinical psychologists. Thus, a younger compared to older age and later compared to earlier years of first hospitalizations are associated with a higher likelihood of reporting utilization of psychotherapy by clinical psychologists. All other associations in this model were either not significant or of little clinical relevance (see odds ratios).

## Discussion

This cross-sectional study surveyed retrospectively self-reported utilization of outpatient psychotherapy among psychiatric inpatients with psychosis and bipolar disorder in a German-speaking part of Switzerland. Results from a reporting period of 5 years indicate that only 23.2% of the participants retrospectively reported utilization (at least one session) of outpatient psychotherapy provided by a clinical psychologist. Furthermore, only 8% to 12.5% of the participants retrospectively reported a recommended amount of psychotherapy for psychosis (at least 16 or 25 sessions, see S3-Guidelines^2^). These already low rates of psychotherapy utilization provided by clinical psychologists were lower among participants diagnosed with F2X compared to F31 disorder. Our results indicate that clinical psychologists played a rather minor role in the outpatient care of people with psychosis and bipolar disorder in this patient sample, especially when the psychologists were part of the outpatient treatment network not associated with the clinic.

These preliminary findings are in line with prior studies indicating underutilization of psychotherapy—especially for people diagnosed with psychosis and bipolar disorder—in the USA, UK, Australia, Germany, and Italy,^6-8,10-12^,^14,18^ see also Burgess-Barr et al^38^ for an international overview. Low utilization rates were replicated in the present sample, even though Switzerland and in particular the catchment area described in this manuscript is known for a comprehensive health care system associated with a high density of inpatient and outpatient health care providers.^23,39^

Prior to recruitment of participants for this study, the inpatient clinic had already implemented systematic measures at the Centre for Psychotic Disorders to increase rates of outpatient psychotherapeutic care among inpatients. These measures included routinely recommending psychotherapy as an outpatient follow-up treatment within discharge management. Additional measures included that all inpatients received an offer to talk to clinical psychologists during their inpatient stay, aiming to familiarize patients with evidence-based psychotherapy concepts and fostering patients’ interest in psychotherapy by identifying their personal treatment goals.^40^ As many participants in this survey had multiple inpatient stays in the period before their index stay (see Table 2), these measures may have enhanced the utilization of outpatient psychotherapy by clinical psychologists and may further explain why self-reported utilization was primarily present in the last 12 months (vs in the last 5 years). However, retrospectively self-reported utilization remains low in the here presented patient sample.

To better understand associations between patient attributes and reported utilization of outpatient psychotherapy provided by clinical psychologists, we found that the presence of a F2X versus a F31 diagnosis was associated with lower self-reported utilization. In contrast, we detected no significant associations between patients retrospectively self-reported utilization of psychotherapy provided by clinical psychologists and most of their psychological attributes such as attitude toward psychotherapy or hope, self-esteem, and self-efficacy as well as psychiatric attributes such as psychopathology (but see the limitations section below). Should this preliminary finding be replicated in future studies in regards to the present catchment area, this could indicate that it may not be patient characteristics that are responsible for low utilization of outpatient interventions, but rather specific barriers in the health care system (eg, low access to therapies; see Stefanova et al^13^). This hypothesis would also be in line with prior studies suggesting that people with severe psychotic disorders take up evidence-based psychotherapy services when they are accessible and offered^12^ and that they can almost always name treatable intervention targets for which they want treatment,^41^ making it seem unlikely that underutilization can predominantly be explained by patient attributes.

### Limitations

Our survey consisted of a cross-sectional design where we relied on retrospective self-reports to assess the number of psychological psychotherapy sessions over an extended period of 5 years. Eventually, participants might have found it difficult to recall how many sessions they had a few years ago. However, we investigated whether they reported any psychological psychotherapy sessions at all, which may be more easily retrieved from memory. We further collected information about other outpatient therapies during the reporting period to detect potential treatments offered by clinical psychologists that were not remembered as psychotherapy. Nevertheless, the here presented numbers can only serve as rough estimates for actual utilization of psychotherapy provided by clinical psychologists.

Estimates may further be biased, as we focused on the utilization of outpatient psychotherapy, but did not specify whether patients had received evidence-based interventions during these sessions. Prior studies indicated a gap between utilization of psychotherapy sessions and receipt of evidence-based interventions recommended in the current guidelines.^7,42,43^ Consequently, the number of participants who received outpatient evidence-based psychotherapy, which is in line with current recommendations, may be even lower than reported in this manuscript. Participants in our study were, furthermore, inpatients with an F2X or F31 diagnosis seeking treatment in an inpatient clinic. This results in selection effects concerning the sample and we describe above that inpatients were often encouraged to utilize outpatient psychotherapy (see above). Further research, therefore, needs to explore whether the here presented findings may generalize to patients who utilize outpatient treatment only.

We, further, estimate treatment by relying on patients’ self-reported outpatient psychotherapy provided by clinical psychologists. Psychiatrists, however, can also provide evidence-based outpatient psychotherapy, in addition to psychiatric and medical consultations. This, in turn, could lead to an underestimation of the estimated self-reported utilization of outpatient psychotherapy in this study (but see Tadmon and Olfson^26^). However, a self-reported median of about 5 sessions with a psychiatrist in the past year may indicate that most patients did not receive recommended levels of evidence-based outpatient psychotherapy.

The cross-sectional design, moreover, might be considered a caveat of our study: Our exploratory bivariate regression models can only investigate associations and not temporally or causally directed effects between variables. Assessing attitudes (or other variables subject to change over 5 years) toward psychotherapy during the inpatient stay might, moreover, not be associated with seeking treatment in the previous years, therefore limiting the size of potential effects we were able to detect in this study. Although we consider it a strength of this manuscript that we focus on an inpatient group with severe psychotic disorders, the sample size of this survey is rather small and contains only a small proportion of participants who reported the critical signal (utilization of psychotherapy) we were interested in. This results in low statistical power to detect associations. Future research may therefore aim to conduct a well-powered longitudinal study, assessing antecedents and consequences of utilizing psychological psychotherapy among a larger number of patients with a F2X or F31 diagnosis.

### Potential implications for practice

The preliminary findings from this survey need to be replicated before clinical implications may be derived. Nevertheless, the results of our study showed that only a small subgroup of patients with psychosis and bipolar disorder retrospectively reported having utilized a number of outpatient psychotherapy sessions provided by clinical psychologists. As discussed above, many of the patients in the survey had been readmitted to the inpatient clinic multiple times although they were assisted in finding an outpatient support network before their discharge. However, barriers to outpatient psychological interventions still seem to be present. These may include limited resources at outpatient treatment services^6^ and a requirement of more evidence-based training options in psychotherapy for psychosis and quality supervision for psychotherapists,^16^ who may not be working with patients with psychosis or bipolar disorder yet and might feel insecure in doing so. An increase in resources would, nevertheless, be a decision made by the government and health system, and an increase in training options in psychotherapy would be the responsibility of the respective universities and training institutes providing the postgraduate training.

## Conclusion

Evidence-based psychotherapy for patients with psychosis and bipolar disorder is effective and recommended in current treatment guidelines. Our study indicates, however, that retrospectively self-reported utilized numbers of previous outpatient psychotherapy sessions provided by clinical psychologists in the last 5 years are low among surveyed patients with psychosis and bipolar disorder staying at a Swiss inpatient psychiatric clinic. These findings are in line with prior studies reporting a substantial undersupply and underutilization of outpatient psychotherapy in Germany, Italy, the UK, and the USA. If preliminary findings of this study are replicated, an increase in offers and delivery of evidence-based outpatient psychotherapy for psychosis and bipolar disorder is recommended in the catchment area of this study. This would increase the likelihood that all people with the respective diagnosis are able to access evidence-based therapy.
